# The research output of rod-cone dystrophy genetics

**DOI:** 10.1186/s13023-022-02318-5

**Published:** 2022-04-23

**Authors:** Lama Jaffal, Zamzam Mrad, Mariam Ibrahim, Ali Salami, Isabelle Audo, Christina Zeitz, Said El Shamieh

**Affiliations:** 1 Rammal Hassan Rammal Research Laboratory, PhyToxE Research Group, Faculty of Sciences, Lebanese University, Nabatieh, Lebanon; 2 Department of Biological and Chemical Sciences, School of Arts and Sciences, Lebanese International University, Beirut, Lebanon; 3 Department of Mathematics, Faculty of Sciences, Lebanese University, Nabatieh, Lebanon; 4 Sorbonne Université, INSERM, CNRS, Institut de La Vision, Paris, France; 5 CHNO Des Quinze-Vingts, INSERM-DGOS CIC1423, Paris, France; 6 University College London Institute of Ophthalmology, London, UK; 7 Department of Medical Laboratory Technology, Faculty of Health Sciences, Beirut Arab University, Beirut, Lebanon

**Keywords:** Rod-cone dystrophy, Research output, Human development index, Next-generation sequencing

## Abstract

Non-syndromic rod-cone dystrophy (RCD) is the most common condition in inherited retinal diseases. The aim of this study was to evaluate the research output and productivity related to RCD genetics per countries as classified by the human development index (HDI), by analyzing publication frequency and citations, the choice of journals and publishers, since 2000 to date. We have also analyzed the use of next-generation sequencing (NGS) in publications originating from countries with different HDIs. One thousand four hundred articles focusing on non-syndromic RCD were downloaded and analyzed. Citations and published articles were adjusted per one million individuals. The research output is significantly higher in very high HDI countries (86% of the total publications and 95% of the citations) than countries with lower HDIs in all aspects. High and medium HDI countries published together 13.6% of the total articles worldwide and received 4.6% of the citations. On the publication level, the USA (26%), United Kingdom (10%), and Japan (7%) were the top 3 among very high HDI countries, while China (6%) and India (2%) ranked first in high and medium HDI countries respectively. On the citation level, similar profiles were found. Following adjustment for population size, Switzerland (~14%), Jordan (~ 1%) and Morocco (<0.2%) showed the highest rates of publications in very high, high and medium HDI countries respectively. Very high HDI countries published 71% of their papers in first quartile journals (first quartile in Scimago journal rank; Q1), and 23% in Q2 journals. High and medium HDI countries showed a similar profile in quartiles with ~ 40% of their papers published in Q1 journals and ~ 30% in Q2 journals. The first publication using NGS was issued in 2009 in very high HDI countries, while it appeared in 2012 in high HDI countries, and in 2017 in medium HDI countries, with a respective lag of 3 to 8 years compared to very high HDI countries. A profound gap exists between very high HDI countries and the rest of the world. To fill it in, we propose implementing NGS, supporting international collaborations, building capacities and infrastructures, improving accessibility of patients to services, and increasing national and international funding.

## Introduction

Rod-cone dystrophy (RCD), also known as retinitis pigmentosa, is an inherited retinal disease (IRD) characterized by the progressive degeneration of the rod and cone photoreceptors [19]. This deterioration results in night blindness followed by progressive centripetal constriction of the visual field in most cases [19]. RCD is transmitted as a Mendelian trait while being exceptionally heterogeneous [37]. At the genotype level, mutations in different genes may cause the same phenotype and numerous disease-causing mutations are reported in each gene [37]. Different mutations in the same gene may cause different clinical consequences [37]. The visual impairment in RCD substantially alters daily functioning and well-being [18], and can have a significant psychological impact on affected individuals, especially those residing in developing countries with limited resources, where this condition is sometimes misdiagnosed.

Genetic counseling through pedigree analysis followed by genetic testing is available to advise the affected individuals, help them adapt, and provide them with the risk of transmitting this condition to their descendants. To expand the current knowledge and uncover disease mechanisms in RCD, many genetic studies reported novel genetic variants and showed substantial ethnic differences, which implies the need for conducting genetic studies all over the globe [8, 9, 25, 39].

The human genome's (HG) sequencing provided RCD (and the inherited retinal degenerations in general) with vast amounts of new causative genetic variants [49]. Although important, the identified genotype–phenotype associations relied on classical techniques that were labor-intensive and time-consuming, including linkage analysis, homozygosity mapping, and Sanger sequencing [6]. Since 2008, next-generation sequencing (NGS) platforms have become widely available, allowing a rapid sequencing of the HG at a reduced cost [43]. NGS technique has become a powerful tool for identifying novel genotype-RCD associations [1, 15, 29]. This breakthrough led to an expansion in RCD's genetic studies, which requires a comprehensive review of the research output and its trends.

Despite all the progress done so far in RCD genetics, there is currently no bibliometric analysis evaluating the research output and productivity worldwide. Therefore, we assessed the publication numbers, citations, publication pattern on the level of publishers and journals, and NGS implementation in this field per country, according to the human development index (HDI) of the United Nations Development Program (https://www.undp.org/), from 2000 to date. Then, we proposed future strategies that might help to bridge the gap between very high HDI countries and the rest of the world.

## Methods

### Search strategy

SES searched and identified all the papers available on Google scholar that contained in their title "rod-cone dystrophy" or "retinitis pigmentosa" since the year 2000 (https://scholar.google.com/, last accessed on November 15, 2020). We only focused on English publications for two reasons: (1) the English publications constitute > 99% of the total publications in Google scholar database and only a very tiny fraction of the identified publications were in non-English (Chinese), and (2) the language barrier does not allow us to read and analyze non-English papers.

### Data extraction, inclusion, and exclusion criteria

SES downloaded all data from the Google scholar database in text format, with all the information such as authors' names, papers' titles, journals' names, publishers, number of citations, and countries of origin. Of the 4132 articles retrieved from Google scholar, 1400 were analyzed. By using the terms "rod-cone dystrophy" or "retinitis pigmentosa" in the initial search, our goal was to be as exhaustive as possible. This search included all the sub-disciplines such as animal models, treatment, gene therapy, stem cells, molecular biology pathways, and genetics. Moreover, this step generated several duplicated articles and non-English articles. Once manual filtering was done by removing all the sub-disciplines except “genetics”, the duplicates, and the non-English articles, 1400 articles were left. Additional information, such as the total population and the HDI of the countries-of-origin, were extracted from the UNDP's Human Development Report (http://hdr.undp.org/en/data), which classified the countries into four categories: very-high, high, medium, and low HDI countries, based on life expectancy, educational attainment, and Gross National Income per capita. ZM added the journals, the publishers, and the Scimago journals' quartiles (SJR: https://www.scimagojr.com/). The SJR is an index of weighted citations per article over three years that gets updates on yearly basis (accessed on December 10^th^, 2020). Citations and documents applied in this formula are based on the Scopus database. The SJR quartiles, Q1 to Q4, refer to journal ranking quartiles within a sub-discipline using the SJR citation index. Thus, Q1 journal has an SJR in the top 25% of journals for at least one of its classified sub-disciplines. SES converted all this data to a tab-delimited file to check for data error. SES and ZM carried out a standardization process of all the included articles.

### Analysis of federal research funds and relation with the research output

LJ and SES downloaded the percentage of the gross domestic product (GDP) spent on research from the Human development reports (http://hdr.undp.org/en/data, 2019). The federal research funds were calculated by multiplying the percentage of GDP spent on research by the total GDP (http://hdr.undp.org/en/data, 2019).

### Analysis of publications using next-generation sequencing data

MI and ZM searched for all the publications having in their title and/or abstract one of the following terms; ‘next-generation sequencing’, ‘whole-exome sequencing’, ‘targeted sequencing’, ‘next-generation sequencing panel’, and ‘whole-genome sequencing'. Following the article identification, ZM and MI independently read the abstract and verified the use of the NGS.

### Statistical analysis

Data were presented as percentages. The number of citations and published papers were adjusted per one million individuals to compare different groups. The chi-squared test was used to evaluate any significant association between Scimago quartiles and the HDI. Spearman correlation analysis was also used to investigate any possible relation between the federal research fund (expressed as % of GDP and total amount) and the research output expressed as publications per million and citations per million. All analysis were performed using SPSS (IBM Corp. Released 2019, SPSS Statistics for Windows Version 26.0, Armonk, NY), and the plots were generated using Origin software (OriginPro, Version 2019b. OriginLab Corporation, Northampton, MA, USA). The level of significance was set at p < 0.05 for all statistical analyses.

## Results

**Fig. 1:**
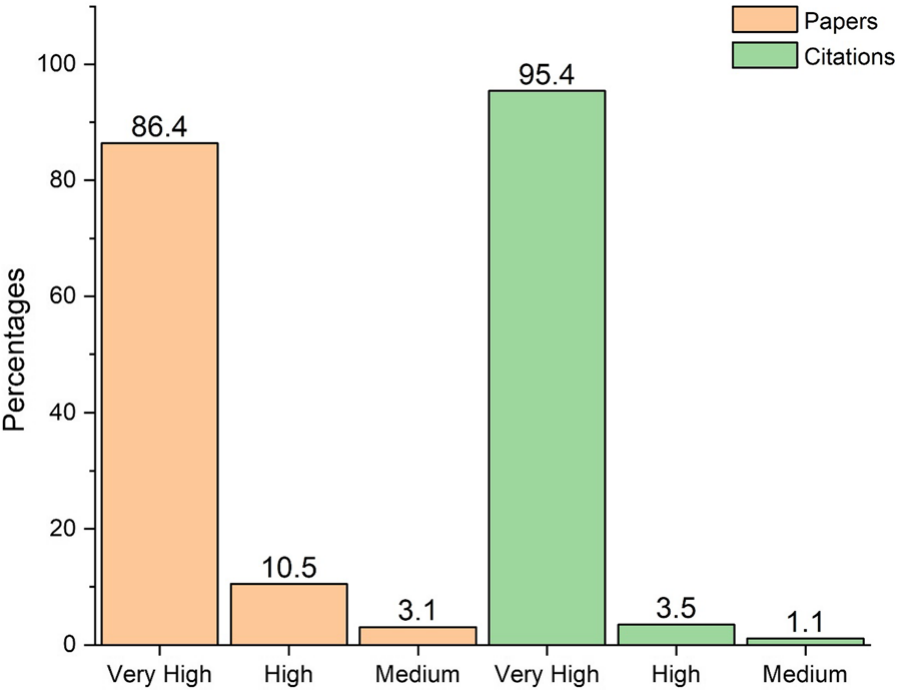
The distribution of published papers and their citations according to the human development index. The papers and their respective citations were adjusted per one million individuals to allow comparison between groups. Data are presented as percentages. The human development index was retrieved from the United Nations Development Program (UNDP, http://hdr.undp.org/en/countries)

**Fig. 2:**
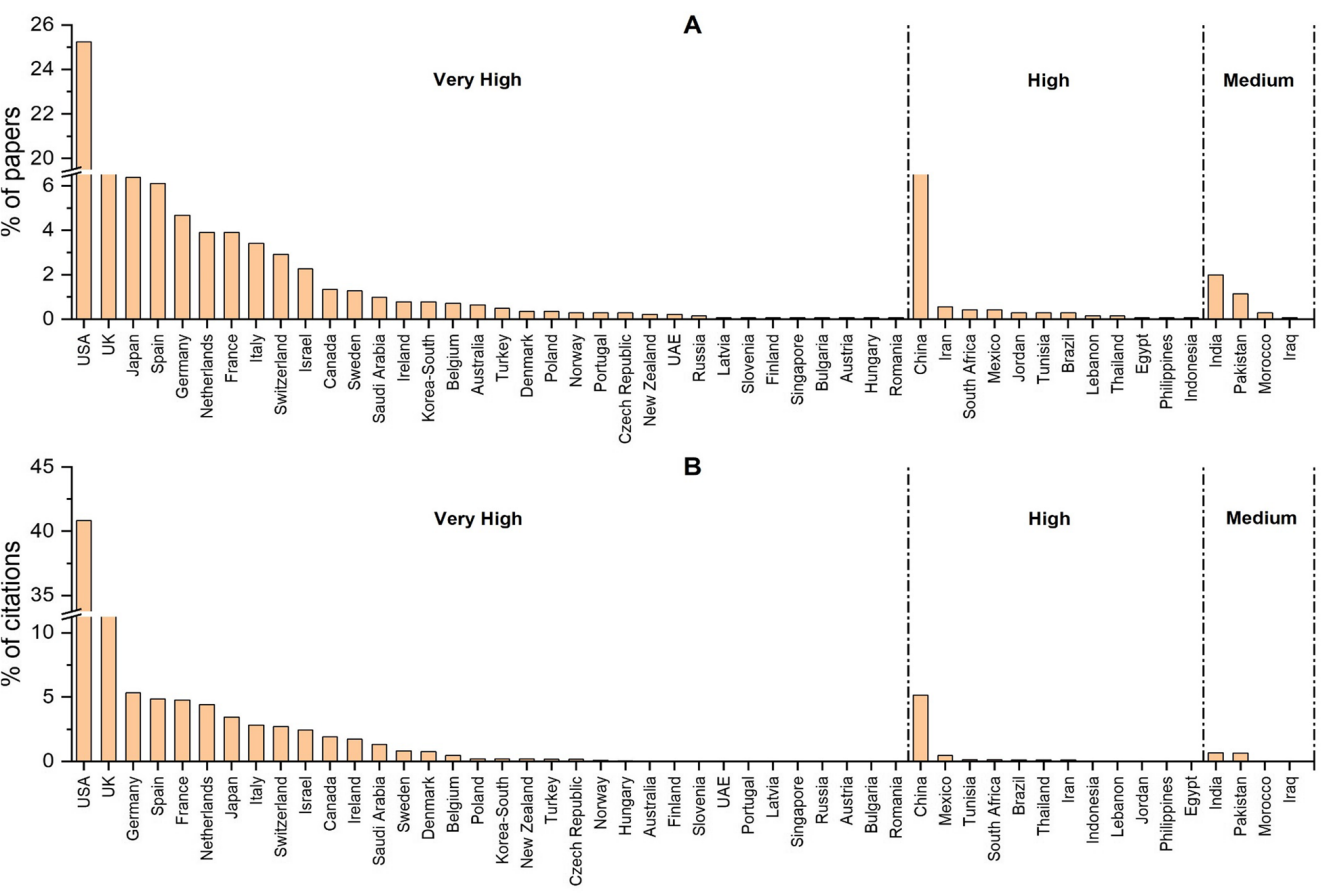
The distribution of published papers and their citations according to countries. The countries are classified based on the human development index of the United Nations Development Program (UNDP, http://hdr.undp.org/en/countries). The papers (**A**) and their respective citations (**B**). %: percentage

Our analysis was conducted on 1400 articles downloaded from Google scholar database. Figure 1 shows the research outcome in terms of papers and citations by HDI. Very high HDI countries produced ~ 86% of the publications and 95% of citations. High and medium HDI countries published ~10% and 3% of the articles, respectively, and got ~3% and 1% of the total citations. In contrast, low HDI countries did not yet publish any paper (Fig. 2).

**Fig. 3:**
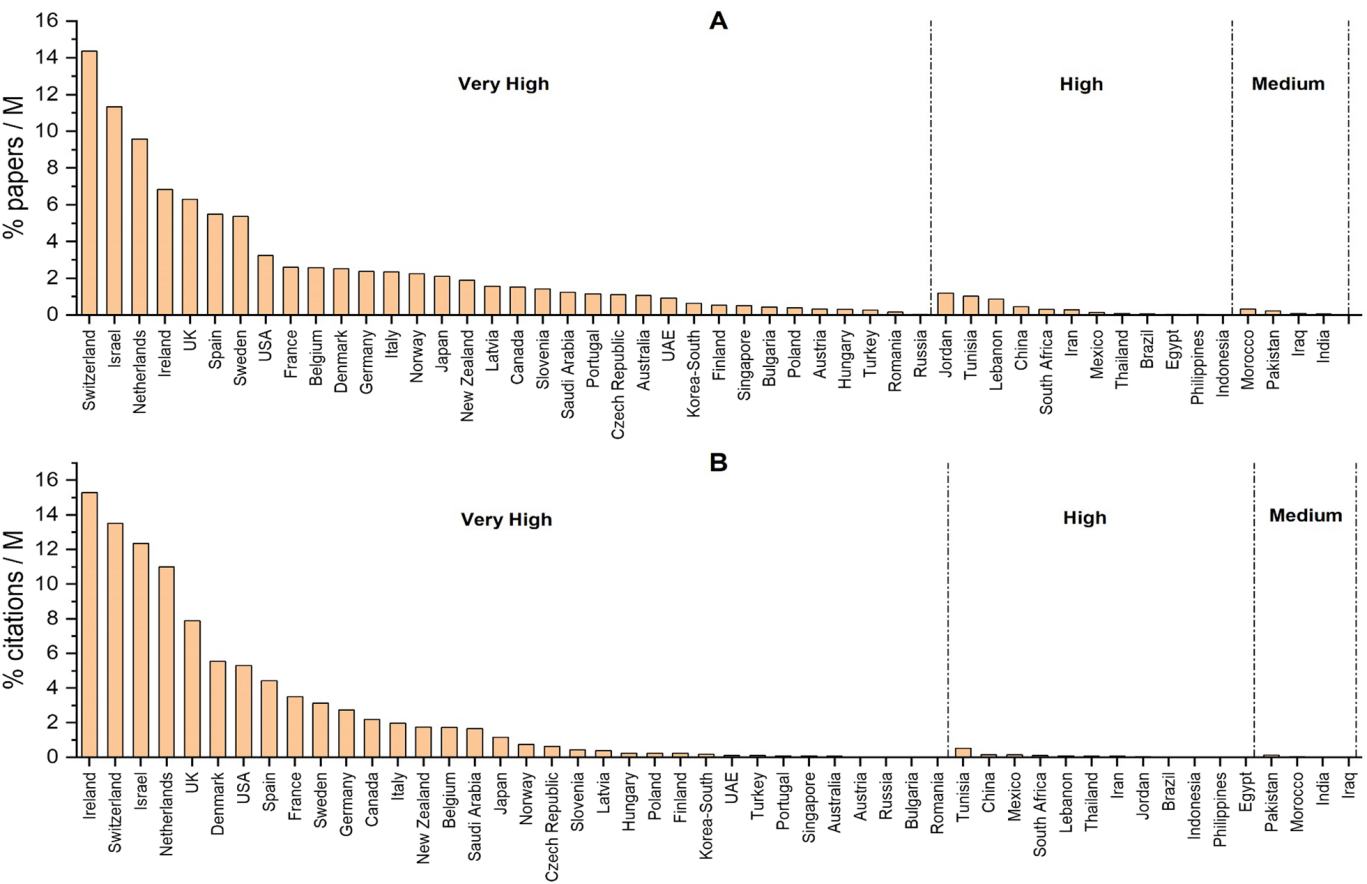
The distribution of published papers and their citations according to countries adjusted per population size. The countries are classified based on the human development index of the United Nations Development Program (UNDP, http://hdr.undp.org/en/countries). The papers (**A**) and their respective citations (**B**) were adjusted per one million individuals. %: percentage, *M*: Million individuals

**Fig. 4:**
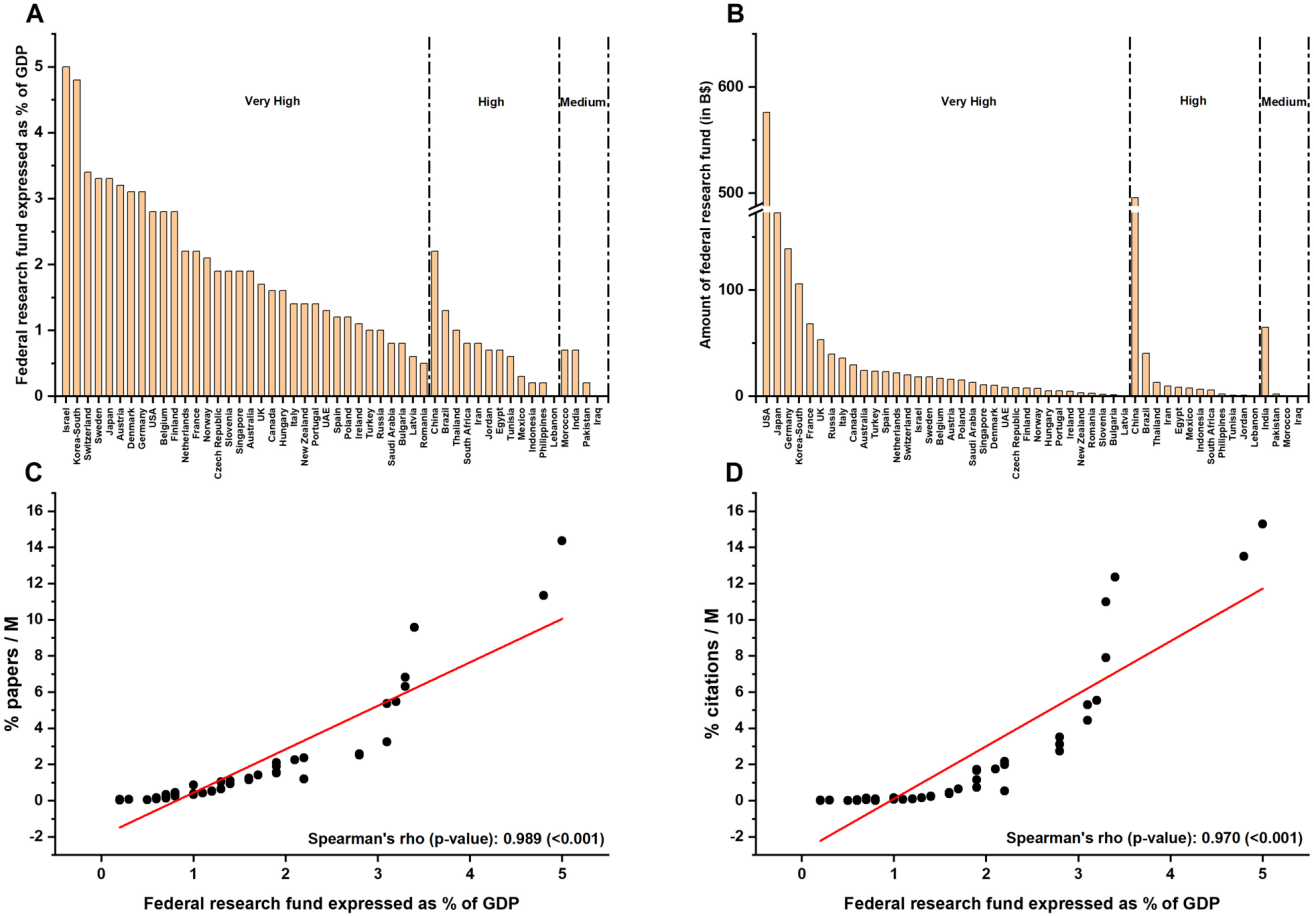
The federal research funds allocated to research and their correlation with the research output. **A** The federal research funds are expressed as a percentage of gross domestic product per country. **B** The amount of federal research funds spent per country. **C** Correlation analysis between the federal research funds and number of publications. **D** Correlation analysis between the federal research funds and the number of citations. The gross domestic product (GDP) values were extracted from the United Nations Development Program (2019). The countries are classified based on the human development index of the United Nations Development Program (UNDP, http://hdr.undp.org/en/countries). The papers and their respective citations were adjusted per one million individuals to allow comparison between groups. *GDP*: Gross domestic product, %: percentage, *M:* Million individuals

On the level of articles, USA (26%), United Kingdom (10%), and Japan (7%) were the top 3 among the very high HDI countries, while China (6%) and India (2%) ranked first in high and medium HDI countries respectively. On the citation levels, similar profiles were seen except that Germany came third (5%). When the papers' distribution was analyzed per country following adjustment per million individuals, we found that the most active ones were: Switzerland, Israel, Netherlands, Ireland, UK, Spain, Sweden, USA, France, Belgium, Denmark and Germany (Fig. 3A). Similarly, the latter countries were also the most cited, except for Belgium (Fig. 3B). Countries with high HDI such as Jordan, Tunisia, Lebanon, and China, showed some research activity in terms of publications but not on the citations' level (Fig. 3A, B). On the other hand, low HDI countries did not show a noticeable output in this field (Fig. 3A, B). Further, we have investigated the percentage of federal funding spent on research in every country and its relation with the adjusted research output (Fig. 4). As shown in Fig. 4, Israel, South Korea, and Switzerland were the top 3 countries in very high HDI countries with 5%, 4.8%, and 3.4% of their GDP, respectively (Fig. 4A). Around 50% of the very high HDI countries spent from 1 to 2% of their GDP on research. In high HDI countries, China and Brazil spent 2.2% and 1.3% of their GDP, respectively (Fig. 4A). In medium HDI countries, India and Morocco had the highest % of federal research funds with 0.7% (Fig. 4A). The amount of federal research funds showed a different profile; the USA, Japan, and Germany were the top three countries in very high HDI countries, while China and India showed the highest amount of federal research funds in high and medium HDI countries, respectively (Fig. 4B). Of interest, the federal research funds were positively correlated with the adjusted number of publications and their respective citations (r > 0.95, P < 0.001). This result shows the absolute correlation (95%) between the adjusted research output and the federal research funds in the field of RCD genetics [31].

In addition to the rates of publications and citations, the Scimago Journal's quartiles were also analyzed and showed that 71% of the papers published by very high HDI countries were in Q1 journals, and 23% were in Q2 journals (Table 1). High and medium HDI countries showed a similar profile in quartiles; ~ 40% of the papers were in Q1 Journals, ~ 30% in Q2, and ~ 24% in Q3 and Q4 journals (Table 1). Medium HDI countries showed the highest rate of publications in non-indexed journals (8%, Table 1).

**Table 1:** Percentage of publications on the genetics of rod-cone dystrophy in indexed journals according to the human development index

HDI category	Quartiles				Not indexed	P
	Q_1_	Q_2_	Q_3_	Q_4_		
Very high (%)	71	23	3	2	1	< 0.001
High (%)	38	33	9	16	4	
Medium (%)	40	28	14	10	8	

Journal quartiles were retrieved from the Scimago database (https://www.scimagojr.com/). Data are presented as percentages. Scimago database was accessed on December 1^st^, 2020. American Journal of Human Genetics, Human Molecular Genetics, JAMA Ophthalmology, Scientific Reports, Clinical Genetics, British Journal of Ophthalmology, Human Genetics and Investigative Ophthalmology and Visual Science are among the Q1 Journals. Ophthalmic Genetics and Molecular Vision are among the Q2 Journals. Current Genomics, Human genome Variation, Molecular Medicine Reports are among the Q3 Journals. Gene reports, Chinese Journal of Medical Genetics, Journal of Genetics are among the Q4 journals

*HDI:* Human Development Index

To go further in our bibliometric analysis, we studied the global publication pattern on the level of publishers and journals (Table 2). About half of the articles of the very high HDI countries were published in Association for Research in Vision and Ophthalmology Inc. (18%), John Wiley & Sons Inc. (13%), Elsevier Inc. (10%), and Taylor and Francis Ltd (8%) (Table 2). Articles from high HDI countries were published in John Wiley & Sons Inc. Molecular Vision, and Nature Publishing group with similar rates (~ 15% each, Table 2). In medium HDI countries, about half of the articles were published in Springer (28%), Molecular Vision (17%), Elsevier Inc. (11%), and the public library of Science (8%) (Table 2).

**Table 2:** The publishers' and journals' distribution in rod-cone dystrophy genetics according to the human development index

	HDI category		
Publisher	Very high	High	Medium
Association for Research in Vision and Ophthalmology Inc	18	5	6
John Wiley & Sons Inc	13	16	–
Elsevier Inc	10	9	11
Taylor and Francis Ltd	8	9	6
Molecular vision	8	15	17
Springer	8	9	28
Nature Publishing Group	7	14	6
BMJ Publishing Group	6	–	6
Oxford University Press	5	2	–
Cell Press	4	–	–
American Medical Association	4	–	3
Lippincott Williams and Wilkins Ltd	3	4	–
Public Library of Science	3	5	8
Academic Press Inc	2	4	6
BioMed Central Ltd	2	5	3
Wolters Kluwer Medknow Publications	–	3	3
*Journal*			
Investigative Ophthalmology and Visual Science	24	9	8
Molecular Vision	11	27	25
Ophthalmic Genetics	10	10	4
Human Mutation	7	5	–
Human Molecular Genetics	7	3	–
JAMA Ophthalmology	5	–	4
American Journal of Human Genetics	5	–	–
Ophthalmology	4	–	–
American Journal of Ophthalmology	4	–	8
Journal of Medical Genetics	4	–	4
PLoS ONE	3	8	13
British Journal of Ophthalmology	3	–	4
Human Genetics	3	5	13
Acta Ophthalmologica	2	5	–
Scientific Reports	2	13	–
Clinical Genetics	2	5	8
Eye	2	7	–
Experimental Eye Research	–	3	8

Only the top publishers and scientific journals were shown. Data are presented as percentages

*HDI:* human development index

The journals' search showed that Investigative Ophthalmology and Visual Science, Molecular Vision, and Ophthalmic Genetics harbored the highest number of publications (55%) for very high HDI countries (Table 2). Top-ranked journals were next to these three journals in the field of RCD genetics, such as Human Mutation, Human Molecular Genetics, JAMA Ophthalmology, and the American Journal of Human Genetics, which is not surprising given their acceptance rate. Molecular Vision, Scientific Reports, and Ophthalmic Genetics were the top 3 journals for high HDI countries (Table 2). While for medium HDI countries, Molecular Vision ranked first (25%), followed by PLoS One and Human Genetics (13% each) (Table 2). No solid conclusion from medium HDI countries’ data can be drawn since their contribution constitutes 3% of the scientific literature.

**Fig. 5:**
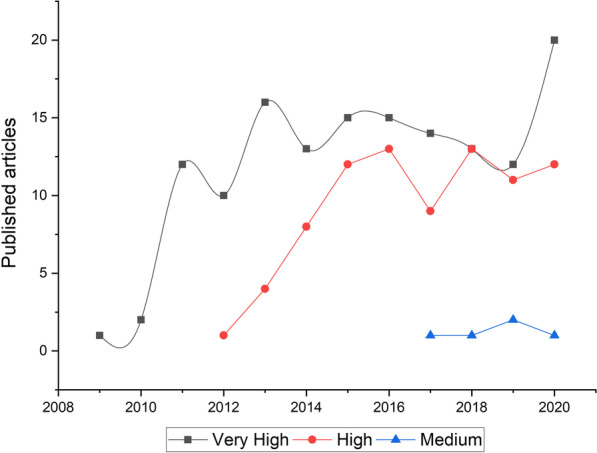
The use of next-generation sequencing in rod-cone dystrophy according to the human development index. The human development index was retrieved from the United Nations Development Program (UNDP, http://hdr.undp.org/en/countries)

Since its invention and implementation, NGS has become a game-changer in genomics by increasing the likelihood of finding novel genotype–phenotype associations and even novel associated genes. The use of NGS in very high HDI countries started in 2009 and kept increasing continuously ever since (Fig. 5). On the other hand, the first publication using NGS in high HDI countries appeared in 2012. Of interest, the curve pattern is similar between very high and high HDI countries (Fig. 5). In medium HDI countries, the first article using NGS appeared in 2017 with a lag of 8 years compared to very high HDI countries (Fig. 5).

## Discussion

Our analysis showed that the research output in RCD genetics is significantly higher in developed countries in all aspects. On the level of articles, USA (26%), United Kingdom (%), and Japan (7%) were the top 3 very high HDI countries, while China (6%) and India (2%) ranked first in high and medium HDI countries respectively (Fig. 2A). On the citation levels, similar profiles were seen except that Germany came third (5%) (Fig. 2B). Following adjustment per million individuals, Switzerland alone was responsible for ~ 14% of the field's publications, while the highest output of the high HDI countries was from Jordan with ~ 1% and the highest production of the medium HDI countries was from Morocco with less than 0.2%. Low HDI countries did not have any detectable research output.

One major factor contributing to those drastic differences is the disproportion in economies and research funds between the countries. Research in most very high HDI countries positively benefits from federal support and private funding agencies, in contrast to countries with fewer incomes. We compared the federal expenditure on research and development (R&D) spent in every country and found out that it was not surprising that Switzerland and Israel showed the highest adjusted research output since they invested the highest percentage of their GDP on research (Fig. 4A). The federal research funds were highly correlated with the number of publications and their respective citations in RCD genetics [31]. Thus, when more funds are provided, the adjusted research output will significantly increase in most countries (Fig. 4C, D).

In addition to limited financial resources, the lack of human resources can be another restraining factor. According to the UNESCO Institute for Statistics, Switzerland (responsible for the highest % of RCD papers in very high HDI countries after adjustment) has 4468 researchers per million inhabitants, while Morocco (responsible for the highest % of RCD papers in medium HDI countries after adjustment) has 1024 researchers per million inhabitants (http://uis.unesco.org/). It is noteworthy that Morocco showed a higher publication rate than Russia and Romania despite the disparity in HDI. Like other North African or Middle Eastern countries, Morocco has a high rate of consanguineous marriages that can reach 19.9–28% of all marriages [3]. Consanguinity cases are estimated to be 59.09% among families with autosomal recessive diseases [3]. Owing to this high rate of consanguinity, the frequency of hereditary genetic diseases is elevated yielding both economic and psychological burdens on societies [3]. Thus, interest in genetic diseases in Morocco is expected. Morocco's research depends to some extent on international collaboration, mainly with France [3]. Some Moroccan genetic centers are partnered with Orphanet (European reference portal network for information on rare diseases and orphan drugs), or INSERM (French National Institute of Health and Medical Research) [3] Moreover, countries with limited resources may prefer to work on genetic research studying DNA, which is less expensive compared to other fields that demand extensive funding.

Another factor that contributes to the low contribution of medium and low HDI countries in research is the limited access of patients to the healthcare service. Of note, physical disabilities, including visual disability, are socially stigmatized in many medium and low HDI countries, preventing people from participating in studies around these diseases and sharing their clinical or familial history for research purposes [23, 33, 47]. Confidentiality concerns are among the constraints that cause patients to worry from being stigmatized, bullied, or socially-unaccepted in case their personal/clinical details were revealed [7]. Our team personally encountered situations where parents worried about their affected children being socially ostracized from making families due to the risk of having affected offspring. Another problem is the lack and/or unaffordability of transport, which prevents access of patients to health centers where the recruitment or other clinical procedures need to be done, especially in the case of disabled patients living in remote rural region [14, 48]. Distrust and misconceptions around researchers, medical practitioners, pharmaceutical companies, governments, and regulations are also among the barriers to participating in research in countries with lower HDIs [7]. This situation complicates the establishment of large cohorts and launching nationwide genetic analysis in those countries, especially in the absence of national registries for patients in most cases.

Another barrier to publishing in the countries with lower HDIs, is the language issue for non-English speaking scientists [5, 30]. Historical reasons that include colonialism, among others, have established English as the lingua franca of academic research worldwide, implying that the convenient use of the English language and rhetoric is an essential factor in the reviewing and rating of a manuscript. Indeed, this decreases the acceptance rate of papers written by non-native English speakers in the high impact journals and impose additional financial problems on authors since the English editing services are not usually included in the article processing charge (APC) [5, 26, 30].

On the level of citations, very high HDI countries that are the most active in publishing RCD genetic studies were also the most-highly-cited with more than 95% of citations. The differences were significant on the citations’ level per country as Ireland alone (the first among very high HDI countries after adjustment) got more than 15% of the citations (Fig. 3B). In comparison, Tunisia (the first among high HDI countries) and Pakistan (the first among medium HDI countries) earned less than 0.5% (Fig. 3B). This can be explained by many factors including that the majority of the papers from very high HDI countries (71%) are published in Q1 journals that are usually the most-cited and visible journals. The rest were in Q2, Q3, and Q4 journals, with a negligible portion in non-indexed journals (1%). In contrast, medium and low HDI countries had fewer papers in Q1 Journals and more documents in the three remaining quartiles. About 13% of their publications are in non-indexed journals that usually have low reader bases and lower-profiles and quality processes, making them unlikely to be cited.

Another factor is that most very high HDI countries papers are issued from prestigious institutions and laboratories that are trustworthy worldwide, which confers them a higher credibility that encourages other authors to cite them rather than citing unrecognizable bodies. In contrast, work from countries with small science systems can be confronted with a stereotyping associated with racial or ethnic biases and may be regarded as having lesser quality [20, 36, 50]. Stereotyping may also affect the peer-reviewing, selection and acceptance/rejection rates of manuscripts because of their countries of origin, as reported in a growing number of papers [5, 21, 30, 50]. The fact that the high and medium HDI countries published together ~ 13.6% of RCD papers but only received ~ 4.6% of the citations suggests that even authors from these countries may prefer to cite the reputable work of very high HDI countries. Of note, a small number of institutions are behind a sizeable fraction of the research output in this field. The UCL Institute of Ophthalmology partnership with Moorfields Eye Hospital is the primary source of publications in RCD genetics in the United Kingdom. In Switzerland, the Institute of Molecular and Clinical Ophthalmology Basel and the Institute for Research in Ophthalmology are responsible for almost all publications in RCD genetics. In France, most papers are published by the *Institut de la Vision* and the Institute for Neurosciences of Montpellier (> 80% of the published papers). All the institutions mentioned above, except the UCL Institute of Ophthalmology (opened in November 1948), are relatively young research centers that bring scientists and clinicians to collaborate and create technological innovations for the benefit of visually impaired patients.

On the other hand, some studies suggest that authors from certain countries prefer to cite national work rather than work from abroad [4]. Thus, it would be expected that the RCD papers of the very high HDI countries receive most of the citations since they largely outnumber the papers from other countries.

The top scientific journals in which the three categories of countries published their papers were all peer-reviewed indexed journals with reasonable acceptance rates on the level of publishers and journals. The first-choice journal in the very high HDI countries was Investigative Ophthalmology and Visual Science that recently became open-access. In high and medium HDI countries, the top journal was Molecular Vision (Molecular Vision publisher) that also ranked second in the very high HDI countries. Remarkably, this journal has no publication charges (although being open-access), permitting the broadest possible visibility at no cost. This might explain its position across all HDI categories, especially those with the lowest incomes that usually face difficulties to pay the publishing fees. Two of the most extensive open-access multidisciplinary mega journals (Scientific Reports and PLoS One), characterized with high acceptance rates and relatively affordable publishing fees, ranked second in high and medium HDI countries, respectively (13% each). Human Genetics (Springer) that also occupied the second rank in medium HDI countries (13%), is characterized by a hybrid publishing model that offers authors the option to publish open or restricted access at no cost. Due to the absence of RCD papers from low HDI countries and the limited output of high and medium HDI countries, we were not able to investigate if APCs constituted a significant barrier to publish open-access in the case of RCD studies specifically. Nevertheless, many studies suggest that APCs may be a barrier to publishing in medium and low HDI countries, although they are offered waivers by some publishers [26, 38].

Our analysis showed that NGS started to appear in publications in 2009 in the very high HDI countries and has kept increasing ever since. This indicates the promptness of their resilient national science systems' to access the latest technologies and provide their researchers with the best equipment. The use of NGS in high HDI countries appeared in publications three years later (in 2012). Then, it increased continuously, indicating that NGS platforms became available in those countries or they got this sequencing service from abroad laboratories. However, the use of NGS in RCD genetic studies was significantly delayed in medium HDI countries. It only appeared in 2017 and remained scarce compared to very-high and high HDI countries, in which NGS witnessed a steady increase after its first appearance (Fig. 5). This is the consequence of the lack in establishing NGS-equipped facilities in those countries, in addition to other obstacles, including the fact that even outsourcing NGS services is not affordable for most local laboratories [22]. Finally, some studies reported that the outsourcing process could result in low-quality outcomes or even fail at any stage [2, 22].

In light of the knowledge now in hand, several actions should be taken to close the existing gap between very high HDI countries and the rest of the world [22]. To do so, we suggest different strategies. 1—Practical implementation of NGS in clinical centers can considerably expand genetic diagnosis of genetically heterogeneous diseases, including IRDs [27]. The utilization of NGS innovations is critical in the most effective health systems worldwide [16]. Remarkable review publications focusing on the impact of NGS on rare diseases have been released [16]. Furthermore, medium HDI countries can implement new sequencing technologies requiring fewer infrastructure demands [22]. Lately, Oxford Nanopore Technologies has launched MinION, the first commercial portable sequencer using nanopore technology [35]. This technology has reduced the demand of the current short-read genome sequencing technologies that require infrastructures established in dedicated sequencing centers to an inexpensive appliance, preparation package, good web connection, and standard personal computer [12, 22]. 2—International biobank networks and data sharing can enhance care and boost RCD research. Through exchanging data, researchers can robustly evaluate the relevance of RCD research findings, expand studies by implementing pooled databases, re-employ data that is difficult to obtain, and pursue novel research horizons. Nevertheless, for biobanks to fulfill their potential objectives, different initiatives have to be implemented to target the barriers that may hinder data sharing between biobanks and researchers (Colledge et al. [11]). Biobanks performance is known to be impeded by the absence of unified data management systems and standardized settings and the existence of different ethical and legal demands among countries [11, 17, 51]). The variation in the operating procedures between different laboratories and nations, including the entire process of sample acquisition, processing, preservation, and storage among biobanks is one of the main challenges that hinder sharing [11]. Hence, samples are not potentially used, as researchers are unlikely to seek non-standardized samples [11]. Therefore, international harmonization is needed to generate standard operating procedures [11, 17). Additionally, not acquiring the proper consent constitutes an ethical issue limiting sample and data usage in research [11]. It is complex to receive approval for prospective investigations, as it is challenging to notify donors about initiatives that are not yet planned [11]. Thus, further contextualizing and refining of consent are needed. 3—Supporting international collaboration is a crucial driver of any research [28]. Over the past two decades, significant efforts were exerted at European and international levels to promote collaboration in rare disease research [28]. Several promising aspects of assistance may achieve this objective, including such mutual collaboration between rich and poor nations [40]. For example, in 2011, the International Rare Diseases Research Consortium (IRDiRC) was established to promote global research cooperation and funding in rare diseases disciplines [32]. Likewise, the European Retinal Disease Consortium (ERDC), which was launched in 2008, is invigorating collaborations in IRDs by exchanging genetic and clinical data (www.erdc.info). Since their launch, numerous joint papers have been issued from this collaboration (www.erdc.info/index.php/papers). Of note, points 2 and 3 are interconnected in many ways. 4—A higher priority needs to be given for building capacities and infrastructures in genomics research in medium and low HDI countries through: (a) advocating researchers' participation in worldwide programs symposiums, (b) supporting their enrollment in the processes of skills and knowledge development, (c) creating genomic data-producing systems with enabling access to these data, (d) establishing bio-sample storage and infrastructure [40]. Furthermore, there is a need to educate and train clinicians on technological systems and lab methods to empower their understanding of scientific bases [13]. However, building and improving infrastructure in these countries is not enough on its own to improve research output since it may be confronted by the problem of affordability and accessibility to these services, as mentioned above [14, 50]. Parallel measures should be implemented to overcome these obstacles; for example, these countries should work to provide a good and affordable transportation network, and the centers of health research should not be limited to urban areas. All of these suggestions will need funding which is a significant issue in these countries, as stated above. Local scientific bodies should play a role and exert efforts in running campaigns that promote interest in science by clarifying the positive research outcomes on a country's socio-economic development to pressure governments and policymakers to increase their expenditure on research and its related infrastructure [41, 42]. Nevertheless, governmental involvement will not be enough to solve the problem. Large international funders like the World Bank should increase their support for building and improving infrastructure [45]. Furthermore, researchers should apply more for international calls for grants and improve their skills in writing good grant or fellowship proposals, increasing the opportunity to get funded [24]. Besides that, securing international funding should be accompanied by a fight against governmental and institutional corruption that is imposing additional threats to this sector [34]. Such corruption cases were reported in multiple medium and low HDI countries receiving global funds; this includes health projects funded through the Global Fund in Uganda, Djibouti, Mali, Mauritania, and Zambia [10, 34] and projects funded by the World Bank in India [44]. Relatedly, concerns were raised around the refusal of the United Nations Development Program, which manages specific grants of the Global Fund, to share its internal audit reports and records [46].

To conclude, a profound gap exists between very high HDI countries and the rest of the world. To fill in, we propose implementing NGS, supporting international collaborations, building capacities and infrastructure, improving accessibility of patients to services, and increasing national and international funding.

## Data Availability

Please contact author for data requests.
